# Managing Patients With COVID-19 in Armenia Using a Remote Monitoring System: Descriptive Study

**DOI:** 10.2196/57703

**Published:** 2024-09-30

**Authors:** Lusine Musheghyan, Nika M Harutyunyan, Abu Sikder, Mark W Reid, Daniel Zhao, Armine Lulejian, James W Dickhoner, Nicole T Andonian, Lusine Aslanyan, Varduhi Petrosyan, Zhanna Sargsyan, Shant Shekherdimian, Alina Dorian, Juan C Espinoza

**Affiliations:** 1 Turpanjian College of Health Sciences American University of Armenia Yerevan Armenia; 2 Department of Medicine David Geffen School of Medicine University of California Los Angeles Los Angeles, CA United States; 3 Innovation Studio Children's Hospital Los Angeles Los Angeles, CA United States; 4 Department of Surgery Children's Hospital Los Angeles Los Angeles, CA United States; 5 New York Medical College Valhalla, NY United States; 6 Department of Population and Public Health Sciences Keck School of Medicine University of Southern California Los Angeles, CA United States; 7 Allied Anesthesia Medical Group, Inc Santa Ana, CA United States; 8 Department of Surgery David Geffen School of Medicine University of California Los Angeles Los Angeles, CA United States; 9 Fielding School of Public Health University of California Los Angeles Los Angeles, CA United States; 10 Stanley Manne Children’s Research Institute Ann & Robert H. Lurie Children’s Hospital of Chicago Chicago, IL United States

**Keywords:** COVID-19, remote patient monitoring, Armenia, web platform, home oxygen therapy, pandemic, global health care, low and middle-income countries, health care infrastructure, Yerevan, home monitoring, resource-constrained

## Abstract

**Background:**

The COVID-19 pandemic has imposed immense stress on global health care systems, especially in low- and middle-income countries (LMICs). Armenia, a middle-income country in the Caucasus region, contended with the pandemic and a concurrent war, resulting in significant demand on its already strained health care infrastructure. The COVID@home program was a multi-institution, international collaboration to address critical hospital bed shortages by implementing a home-based oxygen therapy and remote monitoring program.

**Objective:**

The objective of this study was to describe the program protocol and clinical outcomes of implementing an early discharge program in Armenia through a collaboration of partner institutions, which can inform the future implementation of COVID-19 remote home monitoring programs, particularly in LMICs or low-resource settings.

**Methods:**

Seven hospitals in Yerevan participated in the COVID@home program. A web app based on OpenMRS was developed to facilitate data capture and care coordination. Patients meeting eligibility criteria were enrolled during hospitalization and monitored daily while on oxygen at home. Program evaluation relied on data extraction from (1) eligibility and enrollment forms, (2) daily monitoring forms, and (3) discharge forms.

**Results:**

Over 11 months, 439 patients were screened, and 221 patients were managed and discharged. Around 94% (n=208) of participants safely discontinued oxygen therapy at home, with a median home monitoring duration of 26 (IQR 15-45 days; mean 32.33, SD 25.29) days. Women (median 28.5, mean 35.25 days) had similar length of stay to men (median 26, mean 32.21 days; *P*=.75). Despite challenges in data collection and entry, the program demonstrated feasibility and safety, with a mortality rate below 1% and low re-admission rate. Opportunities for operational and data quality improvements were identified.

**Conclusions:**

This study contributes practical evidence on the implementation and outcomes of a remote monitoring program in Armenia, offering insights into managing patients with COVID-19 in resource-constrained settings. The COVID@home program’s success provides a model for remote patient care, potentially alleviating strain on health care resources in LMICs. Policymakers can draw from these findings to inform the development of adaptable health care solutions during public health crises, emphasizing the need for innovative approaches in resource-limited environments.

## Introduction

COVID-19 placed unparalleled stress on the capacity of health care systems worldwide, particularly in low-resource settings, often exceeding the ability of the local health care system to respond. Mortality from COVID-19 has exceeded 750 million cases and 6.9 million deaths globally, with a disproportionate impact in low- and middle-income countries (LMICs) [1-4]. The Republic of Armenia, classified as an LMIC by the World Bank [5], registered its first case of COVID-19 on March 1, 2020, and declared a state of emergency, then lockdown on March 6, 2020. In June 2020, the country experienced its first COVID-19 peak. During this time, Armenia dealt with acute shortages of hospital beds and oxygen supply [6,7].

In Armenia, the struggle with the COVID-19 pandemic was compounded on September 27, 2020, with the initiation of a large-scale war by Azerbaijan against the Republic of Artsakh (also referred to as Nagorno-Karabakh), east of Armenia. The conflict displaced thousands of ethnic Armenians leading to an influx of refugees and displaced people into the Republic of Armenia, which challenged adherence to public health recommendations to stymie the spread of COVID-19. Additionally, thousands of deaths and injuries from the war further strained health care resources in Armenia. The daily number of COVID-19 cases increased dramatically after the start of the war by 8-fold [6-8].

The health care systems in the Commonwealth of Independent States, of which Armenia is a member, are particularly brittle. Each of these countries inherited the tenets of the Semashko system after the fall of the Soviet Union. The model is centered on an in-patient delivery model with weak primary care systems and low health service use [9]. In Armenia, patients generally require inpatient admission to receive supplemental oxygen and regular monitoring. Starting in May 2020, the Primary Health Care facilities were involved in the treatment of mild to moderate cases of COVID-19 [10]. Each wave of COVID-19 exhausted the inpatient capacity to meet the population’s health care needs. The lack of hospital beds with the capacity to deliver oxygen to patients forced the development of novel health care delivery models in Armenia and elsewhere [11-14].

During the early response to the COVID-19 pandemic, traditionally conservative health care systems rapidly adopted technology to address unprecedented health care needs [15]. In Italy, a home discharge program with supplemental oxygen was quickly developed in response to the COVID-19 pandemic [16]. Following the model in Italy and elsewhere, in mid-April 2021, our team presented a home health care delivery program to the Armenian Ministry of Health.

The COVID@home program was a collaborative effort between The Ministry of Health of the Republic of Armenia, Operation Armenia at the University of California - Los Angeles, the Turpanjian College of Health Sciences at the American University of Armenia, the Department of Population and Public Health Sciences at the University of Southern California, Children’s Hospital of Los Angeles (CHLA), and the Armenian Eye Care Project. During the COVID-19 pandemic’s second peak in Armenia, the shortage of hospital beds with oxygen delivery capacity became apparent, and the COVID@home program was launched. The team consisted of Internal Medicine physicians, Infectious Disease physicians, pulmonary disease physicians, registered nurses, respiratory technicians, project managers, and public health and research experts.

The design and development of the COVID@home technology infrastructure has been previously described [14]. This study describes the program protocol and clinical outcomes of implementing an early discharge program in Armenia through a collaboration of partner institutions which can inform the future implementation of COVID-19 remote home monitoring programs, particularly in LMICs or low-resource settings.

## Methods

### Setting

Seven hospitals in Yerevan participated in the program. The medical and administrative team serving the enrolled patients of the program was located at the National Burn Center of the Ministry of Health of the Republic of Armenia, which was transformed to serve as a triage center for COVID-19 during the pandemic.

### Program Development

The objective of the COVID@home program was to provide oxygen concentrators, home health visits, and to develop an enrollment algorithm for eligible patients. The government of Armenia expressed support for the program by releasing a decree allowing home oxygen therapy to treat COVID-19. The team developed a number of key programmatic assets: inclusion and exclusion criteria, overall clinical workflow (ie, patient journey), clinical protocols, an equipment management protocol, follow-up strategies, and data collection protocols. These resources were provided in English and translated into Armenian. Additionally, a simple mobile web-based app was built to capture clinical data and coordinate care.

The core development team was composed of 3 clinicians, 2 program administrators, 3 software developers, and 1 project manager, all of whom were based in either Armenia or the US. Fourteen Health care providers (HCPs) used the COVID@home Web Application (CAHWA) to directly update the screening, health monitoring, and discharge forms. Data was extracted from the system on an ad hoc basis.

### Inclusion and Exclusion Criteria

Patients hospitalized with a diagnosis of COVID-19 were enrolled during their hospital stay by COVID@home program trained staff. Although polymerase chain reaction tests were widely available and used in Armenia at this time, the lack of robust laboratory information systems made it difficult to confirm a molecular diagnosis, so the clinical diagnosis by the treating inpatient team was used. Patients included in the program had an oxygen saturation of less than 90% on room air, but were able to achieve oxygen saturation greater than 93% with the addition of a maximum of 10 L of oxygen via nasal cannula. Patients were excluded if they had baseline oxygen requirement, did not fit into the oxygen parameters within the inclusion criteria, and otherwise showed evidence of systemic sepsis, as defined by standardized criteria for hypotension and new onset organ dysfunction.

### Patient Monitoring and Clinical Workflow

A patient was referred into the program at the time of hospitalization having met the abovementioned inclusion and exclusion criteria, and otherwise did not have any other reason for needing inpatient care other than ongoing oxygen requirement. Upon the patient’s written consent to participate in this program, the patient was then transported home via the responding COVID@home team while kept on oxygen therapy during transport. Upon arrival at the patient’s home (day 0 of the program), the responding COVID@home team provided the patient with a portable oxygen device, obtained an initial set of vitals, and titrated oxygen settings to reach a target of 93% saturation in room air. Additionally, the COVID@home team provided in-person education to the patient and their family members on the use of oxygen devices, and what alarming symptoms to observe. Patients were also provided an educational pamphlet describing the procedures, symptoms, and instructions for the use of the oxygen device. On day 1, a member of the response team made telephone contact with the patient to assess oxygen saturation. At this point, branchpoint decisions were made. If the patient’s oxygen saturation remained stable, then the patient continued on the same therapy, to be reassessed in 24 hours, via a phone call. On the other hand, if the patient had decreased oxygen requirements, then the home care provider made a home visit to titrate the oxygen down to a goal of oxygen saturation >93%. If the patient’s oxygen requirement increased, then oxygen was titrated up in 1 L/min aliquot with a goal of 93% oxygen saturation. Finally, if unable to reach 93% saturation with a maximum of 10 L via nasal cannula, the patient was immediately transferred to the inpatient setting for escalation of care.

**Figure 1:**
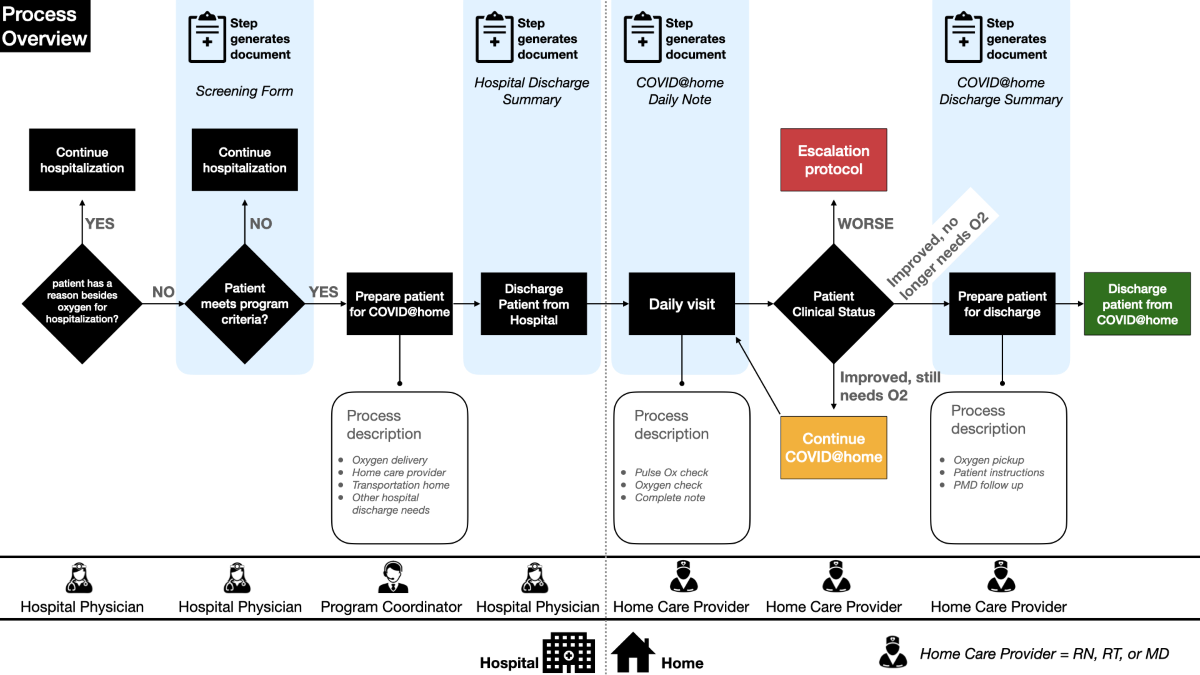
Process map showing the patient journey for patients participating in the COVID@home program. MD: medical doctor; PMD: primary medical doctor; RN: registered nurse, RT: respiratory therapist.

**Figure 2:**
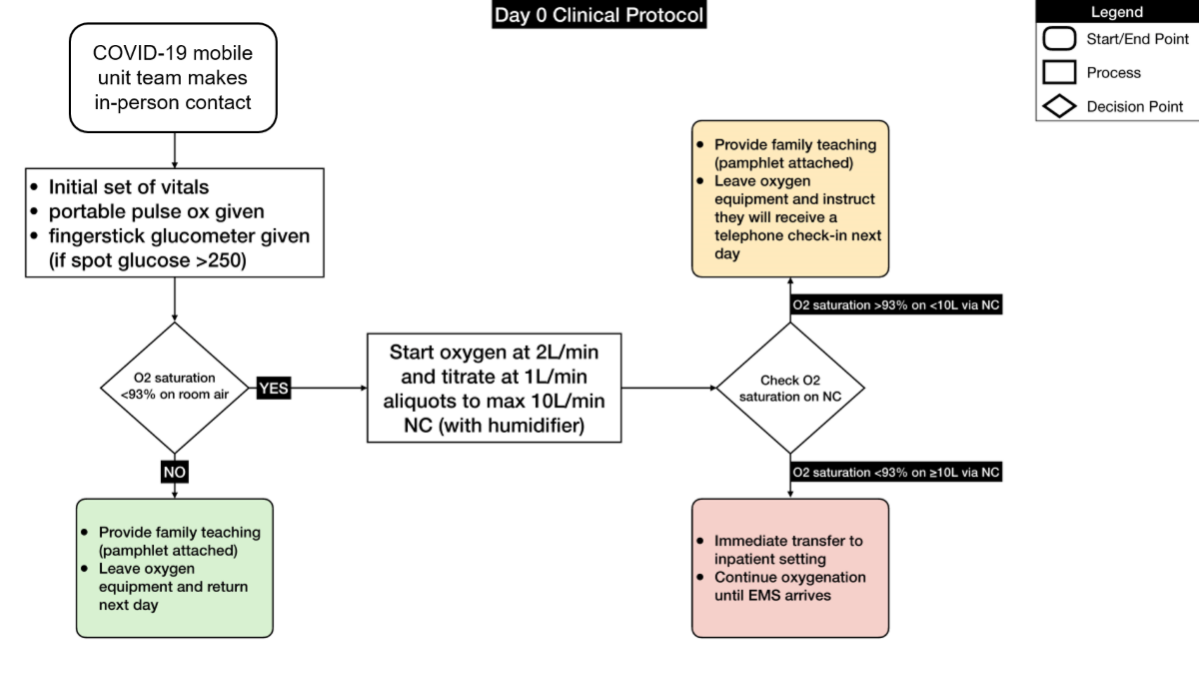
Example of clinical protocols used in COVID@home. EMS: emergency medical services; NC: nasal cannula.

Figure 1 illustrates the overall clinical journey of patients from enrollment to discharge. Figure 2 shows one of the clinical algorithms used for home-based patient management. Clinical parameters were chosen based on current practices in Armenia and the best available evidence at the time from the LitCovid portal [17-19].

### Software Development

The CAHWA development has been previously described in detail [14]. The tool was developed using principles of human-centered design including user-centric functionality, experience, visual appeal, and usability. The software was adapted to the local context through Armenian language support and specific features to improve feasibility. The development lifecycle followed an iterative, agile methodology through weekly development cycles and active feedback from stakeholders [20]. The primary software management tool used was Jira (Atlassian) which stored tickets and documentation and connected to the codebase.

The underlying software of CAHWA is OpenMRS, a Java-based open-source EHR system [21,22]. The final CAHWA architecture included a presentation layer with HTML + jQuery + other frontend technologies (eg, React), a Service layer with Java, and a Database layer with MySQL. Customized ancillary features were developed as OpenMRS modules using the OpenMRS Application Programming Interface. CAHWA contained 3 main forms: screening, daily monitoring, and discharge.

### Data Collection and Analysis

There were three main data sources for the program evaluation: (1) eligibility/enrollment forms, (2) daily evaluation forms, and (3) discharge forms. Data were entered into all 3 forms by the appropriate team members. When possible, data were entered directly into the CAHWA. When not accessible due to technical issues, staff used paper backups of the forms, which were later manually entered into the respective database. Prior to analysis, the data were deidentified, cleaned, and harmonized. A Python script was used to parse key elements from uncleaned data using RegEx. Illegible or erroneous values were removed if parsing was not possible. Data were analyzed using STATA SE (version 14.2; StataCorp). Descriptive statistics were used to summarize participant and program metrics. Length of stay (LOS), defined as days in the program, was calculated by subtracting the Hospital Discharge Date from the Program Discharge Date. When appropriate, continuous outcomes were compared between groups using Mann-Whitney U Tests, and categorical outcomes were compared using chi-square tests.

### Ethical Considerations

The COVID@home program was sponsored by the Armenian Ministry of Health and approved by the participating hospitals’ leadership for clinical implementation in Armenia. This study evaluating the program was approved by the CHLA institutional review board (IRB# CHLA-22-00028). All patients who participated in the program provided written consent.

### Patient and Public Involvement Statement

Patients or the public were not involved in the design, conduct, reporting, or dissemination plans of our research.

## Results

### Patient Recruitment, Participation, and Disposition

**Figure 3:**
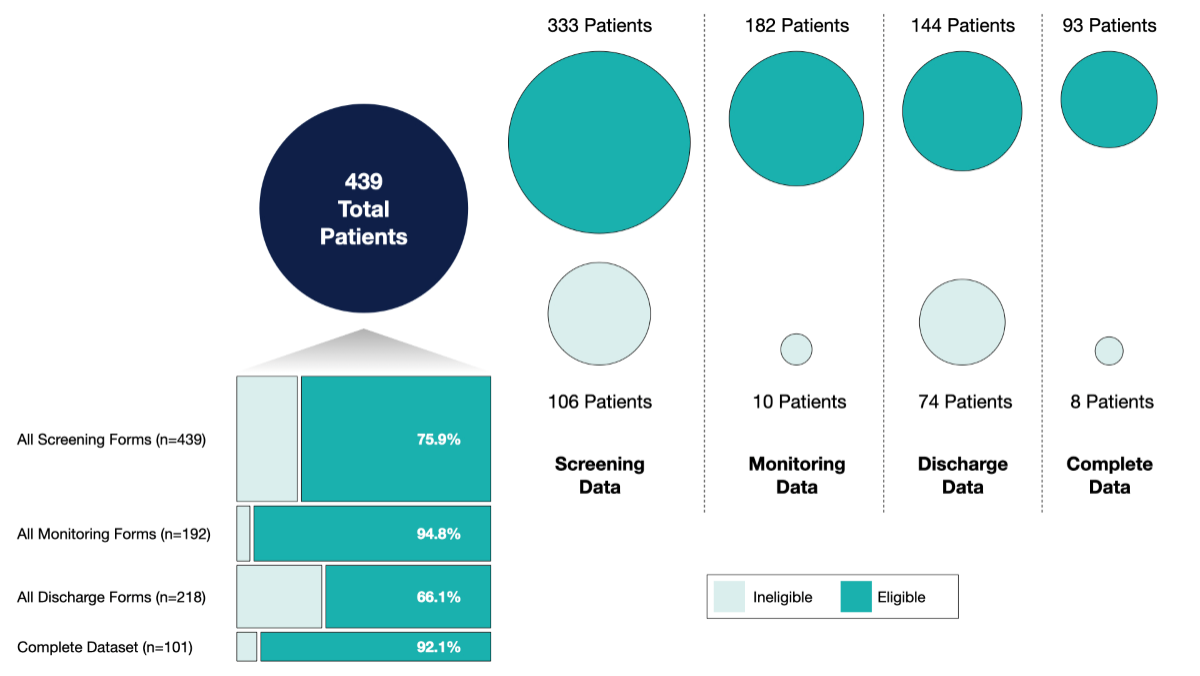
Representation of data availability from each source for both eligible and ineligible patients across their journey through the program.

Between April 2021 and March 2022, there were 439 cases screened for eligibility across 437 unique patients (2 patients were admitted twice during the program), with 75.9% (n=333) meeting eligibility criteria. A number of patients who were deemed “ineligible” were allowed to participate at the discretion of the treating physician and family as a form of comfort or palliative care (data regarding these ineligible patients is presented where available to provide additional context of the situation on the ground and the patients being treated). The main reasons for exclusion were patients not wanting to participate (77/106, 72.6%) and patients exceeding the home oxygen limit (2/106, 1.9%). Three out of the 7 participating hospitals account for over 85% of all patients. Characteristics of the total population, as well as those deemed eligible and ineligible, are shown in Table 1. Figure 3 illustrates patient recruitment, enrollment, and data availability for the COVID@home program.

**Table 1:** Patient screening results.

Characteristics		Eligible patients	Ineligible patients	All patients
Total participants, n (%)		333 (75.9)	106 (24.1)	439 (100)
Female participants, n (%)		170 (51.1)	61 (57.6)	231 (52.6)
Mean age in years (range)		68.3 (0-98)	69.5 (23-90)	68.6 (0-98)
**Originating hospital^a^, n (%)**				
	SGLMC^b^	198 (61.3)	47 (88.7)	245 (65.2)
	MIS^c^	41 (12.7)	5 (9.4)	46 (12.2)
	ACSGLMC^d^	38 (11.8)	—^e^	38 (10.1)
	NCID^f^	36 (11.2)	—	36 (9.6)
	SAMC^g^	9 (2.8)	—	9 (2.4)
	EMC^h^	1 (0.3)	—	1 (0.3)
	SCTO^i^	—	1 (1.9)	1 (0.3)
**Home inspection characteristics, n (%)**				
	Electricity	331 (99.4)	106 (100)	437 (99.5)
	Phone	332 (99.7)	105 (99.1)	437 (99.5)
	Safe environment	332 (99.7)	105 (99.1)	437 (99.5)
Glucose monitor at home		34 (11.3)	0	34 (8.4)
Percentage eligible		—	—	75.8
**Reason for exclusion, n (%)**				
	Declined to participate	—	77 (72.3)	—
	Not applicable	—	27 (25.5)	—
	Higher oxygen requirement	—	2 (1.9)	—

^a^Hospitals from which the patients were enrolled in the program.

^b^SGLMC: Surb Grigor Lusavorich Medical Center.

^c^MIS: Mikaelyan Institute of Surgery.

^d^ACSGLMC: Avan Clinic of Surb Grigor Lusavorich Medical Center.

^e^Not applicable.

^f^NCID: National Center for Infectious Diseases.

^g^SAMC: Surb Astvatsamayr Medical Center.

^h^EMC: Erebuni Medical Center.

^i^SCTO: Scientific Center of Traumatology and Orthopedics.

### Home Monitoring

In total, there were 4430 daily monitoring records across 192 cases; 182 (84.8%) were eligible and 10 (5.2%) were ineligible (Figure 3). Women were approximately half of all patients screened and deemed eligible but had significantly fewer monitoring data; 55 (28.7%) women had monitoring data compared with 137 (71.4%) men (*P*<.001).

The overall rate of vitals documentation varied depending on the metric. Patient pulse was documented most frequently and was found in 4370 out of 4430 monitoring records (98.6%). Oxygen saturation was documented nearly as frequently, in 4234 (95.6%) records. Blood pressure was documented in approximately one-quarter of all records (1121/4430, 25.3%). Documented least frequently, glucose level was noted in 265 (6%) records, and respiratory rate was only noted in 39 (0.9%) records. Out of 192 patients with monitoring data, everyone had Oxygen saturation documented at least once, 190 (99%) patients had pulse documented at least once, and 162 (84.4%) patients had blood pressure documented at least once. Forty (20.8%) patients had their blood glucose checked, and 26 (13.5%) patients had their respiration rate recorded.

### Program Discharge and Clinical Outcomes

Nearly half of the cases screened (218/437, 49.7%) had a discharge form completed. Women had fewer discharge forms completed than men (women: 84/218, 38.5% completed; men: 134/218, 61.5% completed; *P*<.001). Out of 218 patients with discharge data, 205 (94%) discontinued oxygen therapy and were able to be discharged from the program, 7 (3.2%) required rehospitalization, and 5 (2.3%) patients were documented as “stay in program” but did not have a subsequent discharge note. One patient, a 65-year-old male with cancer, died during the program, but his death was not attributed to COVID-19 infection. He was allowed to participate in the program as palliative care and was monitored for most of the days that he was in the program (51 out of 62 days), including the day before he died of cancer-related complications. Due to data collection issues (see Discussion), LOS, defined here as days in the program, could only be calculated for 101 patients. Among those patients, the median LOS in the program was 26 (IQR 15-45; mean 32.33, SD 25.29) days. Women (median 28.5, mean 35.25 days) had similar LOS to men (median 26, mean 32.21 days; *P*=.75).

### Process Metrics and Data Quality Issues

Throughout the implementation of the program, the team faced a number of operational and data quality problems, including missing data, incorrect manual entries, and hardware malfunctions. The primary cause of these data quality issues was the reliance on hard-copy forms when direct entry into CAHWA was not feasible due to sporadic computer and internet access. The limitations of hard-copy forms often resulted in missing or incorrect data, leading to a significant amount of incomplete data.

Specifically, there were significant barriers related to data collection and data entry (see *Discussion* for details), including data missingness, data quality, and discrepancy between patients enrolled/discharged versus patients with data available for monitoring. Around 67 (13.2%) screening records were duplicates or erroneous entries, while another 16 (6.8%) discharge records were duplicates or erroneous entries. Out of 106 patients deemed ineligible at screening, 10 had monitoring data and 74 had discharge data. Among those ineligible patients with discharge data, 66 had discharges recorded on the same date as their screening date.

If a strict definition of study eligibility is applied, 93 (21.2%) patients had a complete data set recorded (screening, at least one monitoring form, and a discharge form). The demographics, LOS, and discharge of those patients are presented in Table 2 in comparison to those of the overall cohort. Among 221 cases without discharge data, 91 (41.2%) cases had monitoring data (89 eligible, 2 ineligible); and among 218 patients with discharge data, 101 (46.3%) patients had monitoring data (93 eligible, 8 ineligible).

**Table 2:** Characteristics of COVID@home participants.

Characteristics		All patients with discharge data	Complete data record
Total participants, n (%)		218 (49.7)	93 (21.2)
Female participants, n (%)		84 (38.5)	4 (4.3)
Mean age in years (range)		67.9 (0-91)	67.7 (27-91)
**Originating hospital^a^, n (%)**			
	SGLMC^b^	109 (50.0)	60 (64.5)
	MIS^c^	27 (12.4)	—^d^
	ACSGLMC^e^	25 (11.5)	17 (18.3)
	NCID^f^	15 (6.9)	11 (11.8)
	SAMC^g^	5 (2.3)	4 (4.3)
	EMC^h^	1 (0.5)	1 (1.1)
	SCTO^i^	1 (0.5)	—
	Unknown	35 (16.1)	—
**Home inspection characteristics, n (%)**			
	Electricity	217 (99.5)	92 (98.9)
	Phone	217 (99.5)	93 (100)
	Safe environment	217 (99.5)	93 (100)
**Glucose meters**			
	Days with monitoring data, average (range)	13.8 (0-99)	29.8 (1-99)
	Percentage with monitoring data	50	100
	Median length of stay (IQR)	26 (3-175)	26 (3-175)
	Percentage zero duration	53.2	3.2
**Outcome, n (%)**			
	Discharged	205 (94.0)	85 (91.4)
	Hospitalized	7 (3.2)	6 (6.5)
	Stay in program	5 (2.3)	1 (1.1)
	Died	1 (0.5)	1 (1.1)

^a^Hospitals from which the patients were enrolled in the program.

^b^SGLMC: Surb Grigor Lusavorich Medical Center.

^c^MIS: Mikaelyan Institute of Surgery.

^d^Not applicable.

^e^ACSGLMC: Avan Clinic of Surb Grigor Lusavorich Medical Center.

^f^NCID: National Center for Infectious Diseases.

^g^SAMC: Surb Astvatsamayr Medical Center.

^h^EMC: Erebuni Medical Center.

^i^SCTO: Scientific Center of Traumatology and Orthopedics.

## Discussion

### Principal Findings

During a challenging moment in Armenia’s history with the rising incidence of COVID-19 cases, shortage of health care resources, and war and its consequences, we aimed to develop a viable remote monitoring program (RMP) to manage patients with COVID-19 at home and reduce the inpatient health care burden. With a team of 14 HCPs, 439 patients were screened for eligibility over the course of 11 months, of which 221 were managed and eventually discharged. Almost all discharged patients (94%) were discontinued from oxygen therapy with an average LOS of 32 days which highlighted the program’s feasibility. Additionally, early discharge was possible even in patients at higher risk as the average age was 68 years. The mortality rate was <1% and only 3% of patients were hospitalized suggesting that the program was also safe. Unanticipated benefits include allowing terminally ill patients to leverage the benefits of CAHWA to improve their quality of life. These promising results emphasize the capacity for home-based care in Armenia and provide evidence of its potential to support patient care.

Based on existing literature, only a limited number of programs have been implemented globally to manage patients with COVID-19 at home with the provision of supplemental oxygen. We identified 8 published studies, 5 of which involved a software app for remote monitoring and 3 relied on more conventional methods such as phone calls [16,23-29]. In one program, providers also visited patients at home to monitor and provide treatment [6]. All the studies were conducted in high-income countries such as the Netherlands (1), the United States (3), the United Kingdom (2), and Italy (1), except for one in Egypt. Although limited, this trend shares the sentiment of existing literature suggesting that LMICs have limited capacity to implement innovation or changes to existing health care systems due to barriers and resource limitations compared with their high-income country counterparts [30-33].

With respect to the clinical protocol, only patients presenting with mild to moderate risk were enrolled in COVID-19 RMPs. This includes a positive reverse transcription polymerase chain reaction test for SARS-CoV-2 and oxygen saturation ranging from 90% to 93% with improving symptoms (eg, patient is apyrexial) but custom risk assessment methods were also used [13,24]. Two studies in the US assessed eligibility for at-home care using proprietary algorithms, which scored patients based on comorbidities, vitals, and demographics. The Atrium Health hospital at-home program assessed patients based on (1) comorbid diseases (hypertension, diabetes, end-stage liver disease, end-stage renal disease, etc), (2) saturation of oxygen (<95%), (3) confusion, (4) respiratory rate ( >22 breaths/min), (5) blood pressure (systolic blood pressure <90 mm Hg or diastolic blood pressure ≤ 60 mm Hg), and (6) age (≥65 years) for a final DSCRB-65 score between 0 and 6. Patients with a score between 3 and 6 with no major signs of pneumonia were deemed as moderate risk and enrolled in the virtual acute care unit on oxygen therapy and monitored remotely. Similarly, the Kaiser Permanente COVID-19 Home Monitoring Program enrolled patients using a COVAS (comorbidities, obesity, vital signs, age, and gender) score of 11-13 considered to be a moderate risk. COVAS assigned 3 maximum points for electrolyte disorders, 2 for BMI ≥40, 7 for saturation of oxygen ≤92%, 3 for age ≥60 years, and 2 for sex as male. Our program operated similarly as only moderate-risk patients with an oxygen saturation of >93% and on <10 L of oxygen via nasal cannula were eligible. In all studies including ours, patients deemed as higher risk at any point of the care continuum were hospitalized and eligible for inpatient services. Similarly, all studies involved ≥2 HCPs including a physician and nurse with the inclusion of other staff members depending on available resources. Patients were contacted by the HCPs over the phone or video at variable intervals depending on the protocol where symptoms and vitals (oxygen saturation, temperature, heart rate, blood pressure) were assessed and modifications were made to care. Generally, in all studies, patients were on <4 L/minute except for the programs in LMICs including ours where the maximum setting was higher (<10 L/min). Patients were discharged from our program on high oxygen due to a shortage of hospital beds and limited resources. Nonetheless, our re-admission rate was low. In other programs, readmissions rates were also low, ranging from 4% to 18%, suggesting that RMPs adequately triage patients and provide reasonable home care. Similarly, mortality rates across all studies were <1% including ours which further highlights the safety of these programs. Study sample sizes ranged from 13 to 147 patients for almost all the studies except 2 conducted at large academic institutions in the United States with sample sizes of 13,055 and 1477 patients. Our program size of 221 patients aligns more closely with the former range which is the majority and therefore more representative of COVID-19 RMPs around the globe. Although inconclusive, the vast discrepancy between large academic institutions in the US and other COVID-19 RMPs is possibly due to financial, structural, and regulatory limitations [34].

Our study’s protocol demonstrated robust outcomes similar to other RMPs, validating its effectiveness despite resource constraints. This critical comparison emphasizes the adaptability and efficacy of our protocol in managing moderate-risk patients with COVID-19 in a resource-limited setting like Armenia.

Strengths of this study include the implementation, approach, and clinical relevance. Despite the multiple challenges, the program was designed and implemented swiftly and collaboratively. The clinical protocol was designed by subject matter experts in both the United States and Armenia and then adapted to fit the local context. Overall, the design and implementation of the program were led by Armenian stakeholders with US collaborators providing support and acting as catalysts. This approach to international collaboration provides a promising model for building partnership and trust, establishing rapport, and executing projects in LMICs which can be challenging [34]. Furthermore, as a real-world clinical solution with a reliable sample size, our program provides practical evidence of remote COVID-19 management.

However, this study is not without limitations. There were many operational and data quality issues such as missing data, incorrect manual entries, and hardware malfunctions [14]. The majority of data quality issues were a direct result of reliance on hard-copy forms when direct entry into CAHWA was not possible due to intermittent computer and internet availability. Due to limitations of hard-copy forms, there was often missing data or erroneous data, which accounts for the relatively large number of incomplete data described in the results. Erroneous data were either corrected or removed from analysis and the clinical staff were provided with remedial solutions by administrators. The significantly higher rate of missing data from female participants is concerning; this could be related to the administrative and operational issues already discussed, or a result of deeper social and cultural biases to be further explored. The results from this program are purely descriptive. There was no control group nor an objective comparator to assess clinical outcomes. At the time of this project, we were also unable to collect comorbidity data, evaluate the cost-effectiveness of the program, or analyze vital trends, which may have provided additional insights into the feasibility and impact of the program. A qualitative study evaluating the clinician experience of COVID@home is underway. Since July 2022, there has been a drastic decrease in patients enrolled in the program as hospitalizations due to COVID-19 have substantially declined and the program is planned to sunset. The Ministry of Health is currently evaluating the utility of this program for other cases.

### Conclusions

In conclusion, RMPs can be implemented to successfully and safely manage mild- and moderate-risk patients with COVID-19 in need of supplemental oxygen therapy despite logistical, economic, and military conflict challenges. However, it requires synergistic effort from a diverse group of subject matter experts. Operational and data quality issues may arise but can be improved through technical and remedial solutions. Overall, the findings from this study were promising and emphasized the capacity for home-based care in Armenia for mild- and moderate-risk patients with COVID-19.

## Abbreviations

CAHWA: COVID@home Web Application
CHLA: Children’s Hospital of Los Angeles
HCP: health care provider
LMIC: low- and middle-income country
LOS: length of stay
RMP: remote monitoring program
